# Family Planning and Preconception Care Service Management: The Key Role of Bulgarian GPs

**DOI:** 10.3390/healthcare12111096

**Published:** 2024-05-27

**Authors:** Eleonora Hristova-Atanasova, Georgi Iskrov, Rumen Stefanov

**Affiliations:** 1 Department of Social Medicine and Public Health, Faculty of Public Health, Medical University of Plovdiv, 4002 Plovdiv, Bulgaria; georgi.iskrov@mu-plovdiv.bg (G.I.); rumen.stefanov@mu-plovdiv.bg (R.S.); 2 Institute for Rare Diseases, 4023 Plovdiv, Bulgaria

**Keywords:** reproduction, women’s health, primary healthcare, general practitioners (GPs), family planning, preconception care, contraception, folic acid

## Abstract

Assisting women in attaining their reproductive goals is crucial for improving the well-being of families and children. As the first point of contact for healthcare, general practitioners (GPs) are ideal for family planning (FP) and preconception care (PCC). However, primary care interventions’ efficacy is unclear. The aim of this study was to examine GPs’ knowledge, attitudes, and perspectives on FP and PCC service management. Most GPs were aware of FP and PCC services and held a firm conviction that they should be primarily accountable together with obstetrician–gynaecologists. However, it is worth noting that less than 50% of respondents reported receiving thorough and comprehensive knowledge of their respective specialities. Those with general medicine qualifications demonstrated a high level of commitment to providing such services. The women’s GPs and those with training in general medicine prescribed birth control pills and emergency contraception three times more frequently than the other doctors who suggested condoms or traditional methods or referred patients to another specialist (*p* < 0.05). In conclusion, PCC is of the utmost importance, and its effective implementation demands the collaboration of policymakers, healthcare providers, and individuals. GPs are essential in managing FP and PCC. They must incorporate more in-depth PCC into their clinical practice.

## 1. Introduction

Assisting women in attaining their reproductive goals and also preventing unwanted pregnancies is crucial for improving the well-being of families and their future generations [1,2]. The period between the third and eighth postconceptional weeks of pregnancy is the most critical for the development of the embryo, and there is a need to fully capitalise on the opportunity to minimise the likelihood of negative outcomes [3,4]. Family planning (FP) and preconception care (PCC) services should provide women with information about risk factors before conception. By doing so, they can take proactive measures as early as possible, ideally before becoming pregnant [5,6].

PCC can be delivered in various settings, either as personalised consultations or as community-based public health programmes targeting all women of reproductive age [7,8]. In certain countries, the integration of PCC into local primary care family planning clinics has been recorded, with general practitioners (GPs) and midwives typically providing these services [5,9,10]. Conversely, several nations have introduced a compulsory preconception counselling system in primary care clinics [11,12]. Healthcare professionals do not consistently address the availability and benefits of FP and PCC in primary care, hospitals, community outreach programmes, and youth health centres, despite the diverse settings in which these settings operate [10,11]. Nevertheless, it has not been consistently provided to all women who may want to conceive, even though the majority of negative pregnancy outcomes occur in women who have not been found to have an elevated risk [13].

Primary care providers are the first point of contact for healthcare, making them ideal for providing PCC. Globally, GPs’ responsibility regarding FP and PCC is of the utmost importance. However, the efficacy of primary care-based interventions is unclear [14,15,16,17]. The utilisation of FP and PCC services is contingent upon GPs’ knowledge, attitudes, and provision of information on PCC to their patients. They can significantly impact couples’ utilisation based on their extensive multidisciplinary skills and knowledge. GPs are responsible for staying up to date with evidence-based clinical practices related to FP and PCC and ensuring they have the most current knowledge to provide preconception counselling [18,19,20,21,22,23].

In many countries, primary care does not prioritise PCC as a standard practice. GPs frequently fail to discuss the accessibility and necessity of PCC [14,24]. The identified obstacles include limited time availability, insufficient knowledge and training, the absence of a reimbursement framework, inadequate coordination and organisation of patient-centred care, and divergent perspectives on professional responsibility [9,18,25].

In Bulgaria, health service packages and standards for FP and PCC consultation are not fully implemented [26]. The provision of FP and PCC services is not considered an essential component of reproductive healthcare, as these activities are formally administered by healthcare providers. The programmes tailored specifically to women of reproductive age (15–49) in Bulgaria primarily focus on addressing reproductive needs, with a particular emphasis on childbearing. The non-stationary sector (GPs and obstetrician–gynaecologists (OBGYNs)) and stationary facilities (specialised hospitals or wards) offer these services, which can be accessed through insurance coverage or paid for privately [27].

Bulgarian GPs are autonomous professionals who operate independently under contract with the National Health Insurance Fund (NHIF) and may conduct individual or group practice. Patients retain the authority to choose their primary healthcare providers [28]. GPs’ administration of FP and PCC interventions to women of childbearing age and the specific measures included in the administration of FP and PCC interventions to women of childbearing age are not clearly defined [29]. Nevertheless, GPs play a pivotal role as the primary healthcare providers in delivering FP and PCC services. Most modern contraceptives do not provide reimbursement, which is especially significant for young and low-income individuals who face challenges in accessing contraception [30,31]. However, the provision of surgical abortion services is free of charge. Conversely, women residing in rural areas of the country have substantial obstacles when attempting to receive specialist healthcare services, with the majority of healthcare services being administered by their primary care physicians [26].

As part of this study, we conducted an in-person survey with GPs in order to examine their knowledge, attitudes, and perspectives on the management of FP and PCC services in primary healthcare practice.

## 2. Materials and Methods

### 2.1. Study Design and Settings

In 2019, we conducted this cross-sectional survey in the Plovdiv region of Bulgaria. The Department of Social Medicine and Public Health at the Medical University of Plovdiv has a longstanding tradition of conducting research on family planning and preconception care. Moreover, the Plovdiv region is a diverse area that consists of significant urban areas, such as Bulgaria’s second-largest city, which is home to multiple advanced healthcare facilities and universities. In contrast, some municipalities in the region depend exclusively on primary healthcare providers. 

We collected primary sociological information using an in-person, self-reported questionnaire in a paper-based format. This study was carried out among GPs in the Plovdiv region in 18 municipalities: Asenovgrad, Brezovo, Hisarya, Kaloyanovo, Karlovo, Krichim, Kuklen, Laki, Parvomay, Perushtitsa, Plovdiv (the administrative centre), Rakovski, Sadovo, Saedinenie, Sopot, Stamboliyski, Maritsa, and Rodopi. The questionnaire was prepared in Bulgarian. On average, filling out the questionnaire took 20 min.

### 2.2. Participants

We collected a purposive sample of GPs who were operating in the Plovdiv region by 1 April 2019. The inclusion criteria were as follows: (1) registration as individual practitioners or as part of a group practice in the Plovdiv region; (2) a valid contract with the NHIF. We identified and invited a total of 420 GPs to participate in the survey.

### 2.3. Questionnaire Design

An original questionnaire was created. The topics were based on research team expertise and on current policies and priorities in the field of family planning and preconception care in Bulgaria [26]. The questionnaire primarily consisted of multiple-choice and single-choice questions. We did not include any questions that required open-ended responses. This study employed an anonymous format and comprised a total of 16 questions across four domains:

1. Sociodemographic profile: The first part of the questionnaire aimed to collect sociodemographic information from the participants, such as their age, gender, medical education, speciality, professional experience, and details about their primary healthcare practice (i.e., the number of patients, the number of women of fertility age, and the number of pregnant women observed).
2. Knowledge and awareness of FP and PC services: This section was designed to collect data on preconception knowledge concerning their speciality, healthcare providers of FP and PCC services, primary prevention activities, and the types of contraception offered in their practice.
3. Attitudes about potential measures to improve FP and PCC services: This section aimed to collect data on potential strategies to enhance the training of general practitioners and the knowledge of women of childbearing age regarding FP and PCC services.

### 2.4. Data Collection

Each GP was visited at their office with a scheduled appointment to fill out the paper questionnaire. After signing the consent form, they were asked to complete the questionnaire immediately. Participation was voluntary, and no incentives were offered. Without assistance from others, each respondent read the questions and independently selected their answers. A pilot study with 10 participants evaluated the survey’s validity and content clarity.

### 2.5. Ethics Approval

Approval by an ethics committee was not required for this study. The survey was sociological from a methodological point of view, with no clinical research. No personal data were saved or analysed.

### 2.6. Data Analysis

The data were analysed using SPSS (version 26.0; SPSS, Inc., Chicago, IL, USA). Figures and charts were generated using Microsoft 365 Office Version 2404 (Build 17531.20152). Quantitative variables were presented as the means ± the standard deviation (SD) of the means. Counts and percentages (*n*, %) were used to report qualitative variables. The chi-square test, one-way analysis of variance (ANOVA) (with a Bonferroni post hoc test), and Pearson’s correlation coefficient test were applied to evaluate statistical differences and associations. The Kolmogorov–Smirnov test checked all numeric variables for normal distributions. For all tests, a level of significance of *p* < 0.05 was adopted.

## 3. Results

### 3.1. Profile of Survey Respondents

A total of 116 (or 27.6%) of the 420 GPs invited to participate completed the entire questionnaire. The mean age of the respondents was 51.5 ± 8.6 years, ranging from 29 to 75. Only 19.0% (*n* = 22) were under the age of 45 years. Women were almost three times more numerous than men.

Only 59.5% (*n* = 69) of the respondents had a speciality in general medicine, with the rest being in the process of specialisation or working without a speciality in the practice of another GP. Most of the respondents had ≥20 years (*n* = 101) of total professional experience, and only 27.6% (*n* = 32) had < 10 years of professional experience in general medical practice. Around 19% (*n* = 22) had a second medical speciality in the field of paediatrics and internal medicine.

### 3.2. Profiles of the GPs’ Primary Healthcare Practices

More than half of the physicians surveyed had primary medical care practices in Plovdiv city. About 81% (*n* = 94) of respondents had up to 2000 patients. Over 2/3 of GPs had fewer than 500 women of fertile age, and only 26.7% (*n* = 31) observed women during normal pregnancy, and they had 40.7 ± 91.6 (*n* = 31) pregnant women. The mean number of women of fertile age had a weak negative correlation with their GP’s age (r_xy_ = −0.28; *p* = 0.003) and professional experience (r_xy_ = −0.28; *p* = 0.003). GPs with an acquired general medicine speciality had a significantly higher mean number of women of fertile age in their practices (F = 5.03; *p* = 0.027) (Table 1).

### 3.3. Knowledge and Awareness of FP and PCC Services

#### 3.3.1. Preconception Knowledge within Their Speciality

During their specialisation, 46.6% (*n* = 54) of the participants made the assessment that their knowledge of FP and PCC was basic and not sufficient. In total, 16.4% of participants (*n* = 19) stated that such concerns had not been taken into consideration, whereas 37.1% of participants (*n* = 43) stated that complete and detailed knowledge had been obtained. The physicians’ learned knowledge did not show any statistically significant difference based on their age and professional experience (*p* > 0.05).

#### 3.3.2. The GPs’ Viewpoints on the Providers of FP and PCC Services

**Figure 1:**
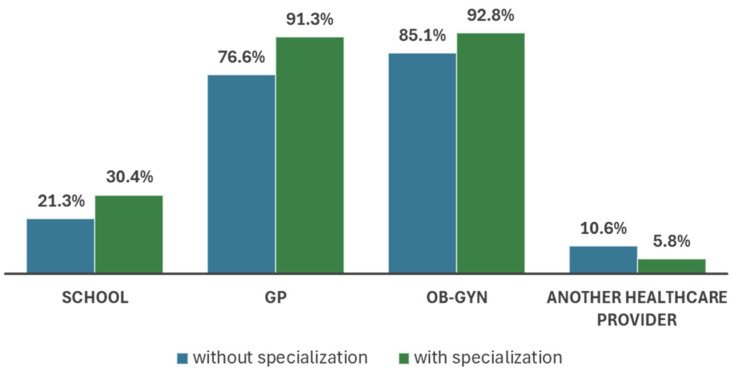
The GPs’ viewpoints on the providers of FP and PCC services.

The respondents strongly believed that OBGYNs (89.7%; *n* = 104) and GPs (85.3%; *n* = 99) should be the main providers of FC and PCC counselling to women. A statistically significant majority of GPs who specialised in general medicine indicated that this was their responsibility, in contrast with those who still did not (χ2 = 8.12; *p* = 0.050) (Figure 1).

#### 3.3.3. Primary Prevention Activities

Healthcare providers should provide every woman intending to conceive with essential information regarding the advantages of folic acid supplementation, as well as the recommended dosages and timing of intake prior to and following conception. The majority of participants (90.5%; *n* = 105) expressed their recommendation for women of reproductive age (15–49) to consume folate prior to pregnancy. Less than half of the participants (44.8%; *n* = 47) suggested taking folic acid 2 months prior, while the remaining participants (28.6%; *n* = 30) recommended intake 1 month before conception or constant use up until conception (26.7%; *n* = 28).

The data reveal that most female GPs offered recommendations regarding folic acid intake, whereas men had a comparatively lower percentage of referrals (χ2 = 6.91; *p* = 0.009). There was a disparity in the guidance provided by male and female GPs regarding the recommended timing of folic acid intake. The advice regarding folic acid intake prior to conception was influenced by the qualifications and accessibility of the general medicine speciality (*p* < 0.05) (Table 2).

#### 3.3.4. GPs’ Awareness of the Types of Contraception Offered in Their Practices

In order to evaluate and analyse the level of knowledge and awareness regarding commonly used contraceptive methods in the GPs’ practices, they were presented with a comprehensive list of contraceptive types. We asked the participants to indicate which types of contraceptives they actively advocated for nulliparous and postpartum women. The findings reveal that a significant number of respondents advocated for using birth control pills and condoms as the primary method of preventing pregnancy.

**Figure 2:**
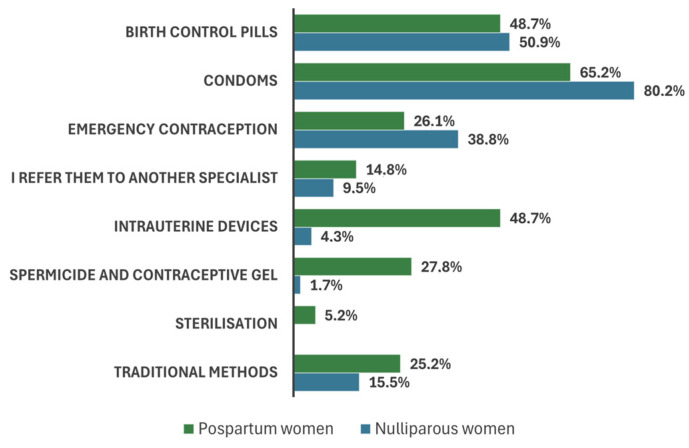
The recommended guidelines for the most appropriate method of contraception based on each woman’s profile (multiple-choice question).

The women’s GPs (χ2 = 22.41; *p* = 0.004) and those with training in general medicine (χ2 = 44.98; *p* = 0.000) prescribed birth control pills and emergency contraception three times more frequently than the other doctors who suggest condoms or traditional methods or referred patients to another specialist. Furthermore, physicians who were over the age of 56 (χ2 = 23.37; *p* = 0.054) and had more than 2000 patients (χ2 = 31.24; *p* = 0.000) tended to refer nulliparous women to another practitioner. The data presented in Figure 2 indicate a difference in the recommended contraceptive methods between postpartum and nulliparous women. The prevalence of condom prescriptions was higher among nulliparous women compared with postpartum women who used intrauterine devices, spermicides, contraceptive gels, and traditional methods (Z = 2.19; *p* = 0.029).

### 3.4. Attitudes about Potential Measures to Improve FP and PCC Services 

Approximately 94% (*n* = 109) of the participants expressed that they would offer and actively distribute information brochures to their patients regarding FP and PCC services. Only 1.7% (*n* = 2) provided a negative response, and 4.3% (*n* = 5) expressed that they would if time permitted. In contrast, a significant proportion of GPs, 75.0% (*n* = 87), were interested in enrolling in a postgraduate programme focusing on preconception care and contraception methods. Notably, the majority of these participants were women (χ2 = 7.32; *p* = 0.026).

## 4. Discussion

### 4.1. FP and PCC Knowledge and Awareness

Population policies primarily focus on promoting population growth and increasing the fertility rate within the specific context of Eastern European countries and Bulgaria. Although Bulgaria has a national programme to improve motherhood and child health [26,32], there has been an apparent lack of primary focus on FP and PCC services in the country’s efforts to increase fertility. Furthermore, FP education and contraceptive methods have not been fully implemented in high schools, despite the fact that they are one of the primary sources of FP and PCC information, alongside family and healthcare providers [33,34]. It is crucial to acknowledge that the mere expansion of programme coverage is inadequate unless all eligible women possess adequate knowledge as well as regular adherence to the appropriate implementation of FP and PCC methods tailored to their specific needs. Raising awareness and knowledge and fostering positive attitudes towards FP and PCC activities among eligible women is strongly advised. Healthcare professionals, especially GPs, must have extensive knowledge and awareness and actively seek advice from women regarding FP and PCC services.

The findings of this study indicate that over 80% of participants were aware of FP and PCC services and held a firm conviction that they should be primarily accountable, together with OBGYNs, for these activities. The data we obtained align with the results of a Dutch study, which demonstrated unanimous agreement among healthcare providers regarding the optimal role of GPs and midwives in delivering PCC consultations [35]. Conversely, other studies indicated that GPs did not perceive themselves as accountable for offering regular pre-pregnancy health and care guidance [5,21,32]. According to Mazza et al., GPs indicated that adhering to PCC guidelines would result in longer consultations compared with standard practices, potentially placing a burden on primary care clinics [36]. GPs frequently encounter time limitations when attending to patients, which pose difficulties in effectively addressing PCC requirements. Insufficient time allocated for patient visits may lead to insufficient conversations on preconception care, resulting in missed chances for evaluating risks, promoting health, and providing counselling [37]. According to Van Voorst et al. [9], the provision of PCC services necessitates sufficient financial resources. GPs in the Netherlands face limited resources to offer preconception care. Additionally, 40% of GPs believed they should bear the responsibility for providing PCC services.

However, it is worth noting that less than 50% of respondents reported receiving thorough and comprehensive knowledge in their respective specialities. Nevertheless, those who possess qualifications in general medicine demonstrated a significant level of engagement in providing such activities (*p* < 0.05). Our study aligns with previous research indicating that GPs possess limited or moderate knowledge and awareness regarding preconception guidelines, necessitating further enhancements. The predominant approach employed by individuals was to depend on knowledge acquired through clinical experience, prior training, and patient information sources rather than relying on established guidelines [21,38,39]. Improving the expertise and abilities of GPs is essential for the efficient delivery of FP and PCC services. By participating in postgraduate education courses, workshops, and conferences and using online resources grounded in evidence-based recommendations and best practices, health professionals can enhance their understanding of developments in PCC. By obtaining the requisite skills, they will be able to provide FP and PCC to all women of reproductive age (15–49) [25,40,41,42].

### 4.2. Primary Prevention Activities 

In accordance with further studies, a significant proportion of fertility-aged women expressed a lack of clarity regarding periconceptional folate supplementation, including concerns related to the optimal dosage, timing, and potential benefits [38,43,44]. In contrast, our study revealed that the majority of GPs administered folic acid prior to conception. However, we observed that not all GPs in Bulgaria consistently demonstrated knowledge of the recommended duration of consumption. Public awareness programmes have been acknowledged as effective means to educate couples and GPs about the need for folic acid consumption throughout the peri-conceptional phase [45]. According to Ray et al.’s review, mass media campaigns promoting the use of folic acid during pregnancy led to a considerable rise in the rate of folic acid supplement consumption, ranging from 1.7 to 7.2 times higher [46]. Furthermore, our analysis aligns with other research that has examined gender disparities in primary care. Several countries have conducted previous research that has identified disparities in examinations, preventative activities, and visit durations between male and female doctors [47,48,49].

### 4.3. Types of Contraception Offered by Healthcare Providers

Bulgaria received an overall score of 55.6% on the European Parliamentary Forum for Sexual and Reproductive Rights Contraception Policy Atlas Europe 2024, which shows that 93% of the 47 countries examined have integrated counselling services into their national healthcare systems [50]. In recent years, several Council of Europe member states have made strides in removing barriers to affordable contraceptive services and information. However, access to a variety of reasonably priced modern contraceptive methods continues to be a barrier for many people in Europe [51]. Only 10% of sexually active women of reproductive age (15–49) who do not want children do not use contraception. With 24% of women using traditional or no contraception (not necessarily due to a lack of alternatives), modern contraception demand is high [32]. Due primarily to the inadequate training of health professionals and the absence of evidence-based public awareness campaigns, numerous member states continue to harbour misunderstandings regarding modern contraception [30,52]. 

In the year 2015, a nationwide survey was conducted. According to the research findings, condoms were the predominant method employed by 90% of the participants to prevent unplanned pregnancies. This was followed by birth control pills at 87% and intrauterine devices at 72%. Many women consulted an OBGYN (58%) or GP (27%) for this purpose [53]. The results of our survey corroborate these findings. A greater proportion of physicians continue to advocate for traditional methods in the prevention of pregnancy. This might be attributed to a deficiency in knowledge, guidelines, or financial incentives that encourage GPs to provide such assistance [54].

The majority of GPs included in our study expressed an interest and actively provided and distributed brochures with information to their patients. This aligns with previous research that has proposed various strategies to enhance the adoption of family planning and preconception care services. These strategies include the distribution of patient brochures, handouts, promotional materials, letters of invitation, posters in waiting rooms and the promotion of PCC-related information through social marketing campaigns [25,36,55,56]. The medical specialists in Bulgaria also use alternative approaches to provide FP and PCC services to various populations. Adolescents with special needs could receive sexual and reproductive health education from the non-governmental Bulgarian Family Planning and Sexual Health Association (BFPA). This organisation aims to promote the provision of mobile reproductive health services in remote and hard-to-reach areas. Using peer-to-peer models, they improved the quality of family planning education in schools [57]. By providing women with information on preconception care, they may make educated decisions and actively seek these treatments to improve their health and the health of their future children [58].

### 4.4. Limitations

There are several limitations to our study’s findings. First, our research focused solely on the region of Plovdiv in Bulgaria, which may restrict the generalizability of our results to the entire country. However, the region of Plovdiv encompasses a diverse range of urban and rural areas, as well as various healthcare providers, making it a fairly representative sample. Therefore, the characteristics of our respondents may align well with the national profile of GPs in Bulgaria.

Second, the relatively low response rate (27.6%) is a significant limitation. This may be attributed to factors such as the busy schedules of GPs and the lack of participation incentives, potentially introducing response bias into our results. GPs with more experience in the subject matter may have been more inclined to participate in the survey.

Third, we relied on self-assessed knowledge from the respondents. However, the high number of GPs expressing motivation to provide FP and PCC services, as well as their willingness to undergo further training, indicates genuine interest and positive attitudes towards this study’s subject.

Finally, we did not address the organisational aspects of FP and PCC service provision in Bulgaria. Exploring implementation factors such as resources, infrastructure, funding, and coordination is essential for planning, implementing, and evaluating any healthcare policy.

## 5. Conclusions

GPs who participated in our survey believed that FP and PCC services should be provided primarily by themselves and OBGYN experts. Although many healthcare practitioners have a positive attitude towards PCC, there is a shortage of knowledge and a need to incorporate PCC into numerous curricula, postgraduate courses, and conferences. Future research should focus on building a new personalised FP and PCC approach that focuses on effectively contacting fertility-age women and physicians using current promotional approaches. Consequently, the findings of this research offer pragmatic insights into the understanding and consciousness of GPs in Bulgaria regarding the FP and PCC services under their supervision. 

In conclusion, PCC is of the utmost importance, and its effective implementation demands the collaboration of policymakers, healthcare providers, and individuals. GPs are essential in managing FP and PCC. They must incorporate more in-depth PCC into their clinical practice. Furthermore, it is imperative that GPs gain extensive knowledge and instruction regarding the importance of PCC, its potential advantages, and the official guidelines that govern its effective execution. This can be accomplished by legislative reforms and additional funding that prioritise the inclusion of FP and PCC services as routine components of reproductive healthcare.

## Figures and Tables

**Table 1:** Sociodemographic and professional profiles.

Profile Characteristic	Overall, % (*n*)
*Gender*	
Male, % (*n*)	29.3% (34)
Female, % (*n*)	70.7% (82)
*Age*	
Age, years (± SD)	51.5 ± 8.6; (range: 29–75)
>45 years old, % (*n*)	19.0% (22)
46–55 years old, % (*n*)	56.0% (65)
<56 years old, % (*n*)	25.0% (29)
*Speciality in general medicine*	
Yes, % (*n*)	59.5% (69)
No, % (*n*)	40.5% (47)
*Other medical speciality*	
No, % (*n*)	81.0% (94)
Paediatrics, % (*n*)	6.9% (8)
Internal medicine, % (*n*)	9.5% (11)
Other, % (*n*)	2.6% (3)
*Professional experience*	
Total professional experience, years (± SD)	25.7 ± 7.9 (range: 5–52)
<20 years, % (*n*)	12.9% (15)
≥20 years, % (*n*)	87.1% (101)
Professional experience in general medical practice, years (± SD)	21.1 ± 9.1 (range: 2–50)
<10 years, % (*n*)	27.6% (32)
≥ 10 years, % (*n*)	72.4% (84)
*Primary healthcare practice*	
*Location*	
Plovdiv city	57.8% (67)
Another municipality	42.2% (49)
Number of patients (± SD)	1478.3 ± 989.0 (range: 350–7000)
<2000 patients	81.0% (94)
≥2000 patients	19.0% (22)
Number of women of fertility age	403.3 ± 989.0 (range: 350–7000)
None	4.3% (5)
<500 patients	65.5% (76)
≥500 patients	30.2% (35)
Number of pregnant women followed up	40.7 ± 91.6 (range: 1–500)
None	73.3% (85)
≤500 pregnant women	26.7% (31)

**Table 2:** The provided guidelines regarding folic acid consumption based on the characteristics of GPs.

Folic Acid Intake		1 Month		2 Months		Constant Use up until Conception		χ2	*p*
		n	%	n	%	n	%		
Gender	Male	15	46.9	7	21.9	10	31.3	11.15	0.004
	Female	15	20.5	40	54.8	18	24.7		
Speciality in General Medicine	With specialisation	12	19.4	30	48.4	20	32.2	6.72	0.035
	Without specialisation	18	41.9	17	39.5	8	18.6		

## Data Availability

The data will be provided upon request.
